# Metatranscriptomics Reveals the Active Bacterial and Eukaryotic Fibrolytic Communities in the Rumen of Dairy Cow Fed a Mixed Diet

**DOI:** 10.3389/fmicb.2017.00067

**Published:** 2017-01-31

**Authors:** Sophie Comtet-Marre, Nicolas Parisot, Pascale Lepercq, Frédérique Chaucheyras-Durand, Pascale Mosoni, Eric Peyretaillade, Ali R. Bayat, Kevin J. Shingfield, Pierre Peyret, Evelyne Forano

**Affiliations:** ^1^UR454 Unité de Microbiologie, INRASaint-Genès-Champanelle, France; ^2^EA4678 CIDAM, Clermont Université, Université d’AuvergneClermont-Ferrand, France; ^3^Lallemand Animal NutritionBlagnac, France; ^4^Nutritional Physiology, Green Technology, Natural Resources Institute Finland (Luke)Jokioinen, Finland; ^5^Institute of Biological, Environmental and Rural Sciences, Aberystwyth UniversityAberystwyth, UK

**Keywords:** rumen, fiber degradation, glycoside hydrolases, carbohydrate esterases, polysaccharide lyases, metatranscriptomics

## Abstract

Ruminants have a unique ability to derive energy from the degradation of plant polysaccharides through the activity of the rumen microbiota. Although this process is well studied *in vitro*, knowledge gaps remain regarding the relative contribution of the microbiota members and enzymes *in vivo*. The present study used RNA-sequencing to reveal both the expression of genes encoding carbohydrate-active enzymes (CAZymes) by the rumen microbiota of a lactating dairy cow and the microorganisms forming the fiber-degrading community. Functional analysis identified 12,237 CAZymes, accounting for 1% of the transcripts. The CAZyme profile was dominated by families GH94 (cellobiose-phosphorylase), GH13 (amylase), GH43 and GH10 (hemicellulases), GH9 and GH48 (cellulases), PL11 (pectinase) as well as GH2 and GH3 (oligosaccharidases). Our data support the pivotal role of the most characterized fibrolytic bacteria (*Prevotella, Ruminocccus* and *Fibrobacter*), and highlight a substantial, although most probably underestimated, contribution of fungi and ciliate protozoa to polysaccharide degradation. Particularly these results may motivate further exploration of the role and the functions of protozoa in the rumen. Moreover, an important part of the fibrolytic bacterial community remains to be characterized since one third of the CAZyme transcripts originated from distantly related strains. These findings are used to highlight limitations of current metatranscriptomics approaches to understand the functional rumen microbial community and opportunities to circumvent them.

## Introduction

The rumen harbors an amazing diversity of microorganisms, comprising prokaryotes (bacteria, archaea) and eukaryotes (protozoa, fungi), which cover essential functions for their host. Part of these microorganisms are specialized in the degradation of plant polysaccharides and thereby constitute a pivotal community providing a supply of energy to the host animal (Hobson and Stewart, 1997; White et al., 2014). Overall, the genomes of fibrolytic microorganisms harbor 100s of genes encoding carbohydrate active enzymes (CAZymes) (Berg Miller et al., 2009; Purushe et al., 2010; Suen et al., 2011; Youssef et al., 2013), mainly glycoside hydrolases (GH), carbohydrate esterases (CE), and polysaccharide lyases (PL) which act synergistically to deconstruct dietary cellulose, hemicellulose, starch, and pectin (Cantarel et al., 2009; Flint et al., 2012). *Fibrobacter succinogenes, Ruminococcus flavefaciens*, and *Ruminococcus albus* are among the first cellulolytic bacteria isolated from the rumen and have long been considered to play a major role in fiber degradation due to their prevalence in ruminants and ability to solubilize efficiently plant cell wall material *in vitro* (Kobayashi et al., 2008; Flint et al., 2012). Nevertheless, recent efforts to isolating novel rumen cellulolytic bacteria (Cai et al., 2010; Dodd et al., 2011; Nyonyo et al., 2014; Ziemer, 2014) and analysis of the diversity of bacterial CAZymes through metagenomic studies (Brulc et al., 2009; Hess et al., 2011; Wang et al., 2013), have provided evidence that bacteria, other than the three most extensively studied, are also involved. Bacteria are usually considered as the main fibrolytic microorganisms in the rumen because of their predominance in this ecosystem. In contrast, rumen fungi are thought to be only minor contributors to plant degradation because of a low biomass in the rumen, although they produce enzymes with a very high specific activity (Wilson and Wood, 1992; Orpin and Joblin, 1997). Protozoa are not essential for survival of the host animal but their removal from the rumen of sheep and cattle has been shown to lower feed degradation in the rumen and decrease feed conversion efficiency in several studies (Newbold et al., 2015). Nonetheless, the mode of action remains unclear as protozoa may contribute directly through the secretion of fibrolytic enzymes or indirectly by creating favorable conditions for fibrolytic bacteria in the rumen (Jouany, 2006). Currently, metatranscriptomics is considered a reliable approach for investigating metabolically active microbial communities which are not necessarily the dominant ones (Torsvik and Øvreås, 2002). The first published rumen metatranscriptomic study was restricted to the eukaryotic community from the muskoxen rumen (Qi et al., 2011). Recently, Dai et al. (2015) and Shinkai et al. (2016) conducted a metatranscriptomic survey of the fiber-attached microorganisms in the rumen of cattle confirming the active fibrolytic status of well-known dominant bacterial degraders. Nevertheless, the relative contribution of eukaryotes to ruminal fiber degradation was not investigated extensively in these studies.

In the present study, RNA sequencing was used to provide a unique insight into the contribution of metabolically active rumen microorganisms to the degradation of four plant polysaccharides (cellulose, hemicellulose, pectin and starch) in the rumen of a lactating cow fed a mixed diet representative of commercial farms. The main objective of this study was to decipher the expression of genes encoding CAZymes to understand better how the fibrolytic community acts in the rumen *in vivo* and to identify potential microbial contributors to this pivotal function. RNA was isolated from a sample of total rumen contents, rather than solids, to investigate both fiber-attached and free floating planktonic population. Compared with previous metatranscriptomic studies (Shinkai et al., 2016), the present work provides a detailed analysis of both eukaryotes and prokaryotes to the CAZyme transcripts. In addition to CAZyme transcripts, we also retrieved sequences known to be involved in fiber-degrading systems such as cellulosomes or Polysaccharide Utilization Loci (PUL; Flint et al., 2012). Data generated were used to highlight some of the limitations of high-throughput approaches to investigate the activity of the rumen microbial population and opportunities to circumvent them based on sequencing of total RNA and a mRNA-enriched RNA fraction obtained using an in-house procedure. Taxonomic analysis of the rumen microbiota was based on small subunit (ssu) rRNA from the total RNA, followed by functional and taxonomic annotation of putative mRNA from the mRNA-enriched RNA fraction. Our results show that the well-characterized CAZyme families and fibrolytic bacteria are the major contributors to polysaccharide degradation in the rumen of a cow fed a mixed diet, but also underscored that the contribution of eukaryotes is underestimated.

## Results and Discussion

### Insight into the Active Rumen Community Based on rRNA and Putative mRNA

**FIGURE 1:**
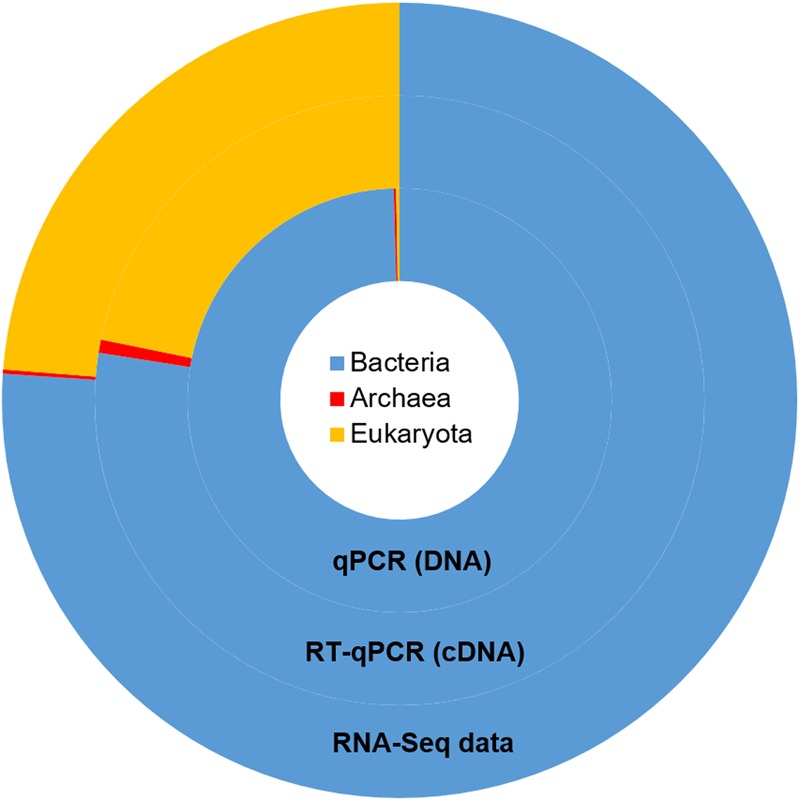
**Taxonomic repartition of the active population of the rumen microbial community derived from rRNA sequences from the metatranscriptomic data (outer ring) and the relative abundances of main microbial groups as measured by qPCR at the DNA and RNA level (inner rings).** The same color code is used for both data.

Taxonomic mining of microbial communities by small subunit rRNA analysis has been widely applied to decipher active members. Here we used 16S and 18S rRNA sequences from the total RNA sample to highlight the active prokaryotes (bacteria and archaea) and eukaryotes (protozoa and fungi) of the rumen. Bacteria represented the majority of the rumen ssu rRNA reads (77.5%; **Figure 1**). They exhibited a high diversity that included 23 identified phyla (Supplementary Table S1) of which only 6 were detected at a relative abundance above 1% (i.e., Firmicutes, Bacteroidetes, Fibrobacteres, Proteobacteria, Spirochaetae, and Lentisphaerae; **Table 1**). While Li et al. (2016) found the phylum Proteobacteria representing between 5 and 90% of bacteria ssu in total RNA, it represented only 5.5% in the animal studied here. At the family level, *Prevotellaceae* (20%), *Ruminococcaceae* (13.2%), *Lachnospiraceae* (9.1%), and *Fibrobacteraceae* (6.8%) were the most abundant in the bacterial population (**Table 1**). While archaea contributed to only 0.7% of total ssu reads, eukaryotes accounted for 21.8% (**Table 1**). Sequences from eukaryotes were dominated by the Intramacronucleata subphylum (90.4% of the eukaryota) associated to the rumen ciliate protozoa and the Neocallimastigomycota phylum (3.3%) corresponding to the rumen anaerobic fungi (**Table 1**). Other eukaryotic families pertained to the phylum Excavata regrouping some flagellate protozoa (Hampl et al., 2009) for which the commensal status is not confirmed because none have been extensively characterized (Fonty and Chaucheyras-Durand, 2007). The high relative contribution of the Intramacronucleata subphylum to the ssu rRNA reads, confirmed by RT-qPCR, contrasts with its low relative abundance on a DNA basis (**Table 2**), leading to a ssu RNA:DNA ratio of 542. This suggests that protozoa were very active in the rumen of cow under the specified conditions of this experiment (diet containing 50:50 grass silage:concentrates on a dry matter basis and sampling of rumen contents before morning feeding). Nevertheless, this finding cannot be confirmed using taxonomic binning of non-rRNA reads, where putative mRNA of rumen protozoa accounted for only 0.70%. Low database completion may bias our overall view of the active microbiome as few reference genomes and genes from rumen eukaryotes are currently available. Indeed no rumen protozoal genome and only one fungal genome (*Orpinomyces* sp. C1A, Youssef et al., 2013) are available.

**Table 1:** Taxonomic representation of the active rumen microbiota based on small subunit (ssu) rRNA sequence analysis from RNA-sequencing data obtained with total RNA.

Superkingdom	Phylum	Family/Subphylum
Bacteria (77.5%)	Firmicutes (33.2%)	*Ruminococcaceae* (13.2%) *Lachnospiraceae* (9.1%) *Christensenellaceae* (4.8%)
	Bacteroidetes (30.1%)	*Prevotellaceae* (20.0%) BS11 gut group (1.7%) *Rikenellaceae* (1.6%) Unclassified Bacteroidetes (1.7%) RF16 group (1.2%)
	Fibrobacteres (6.8%)	*Fibrobacteraceae* (6.8%)
	Proteobacteria (5.5%)	*Succinivibrionaceae* (4.3%)
	Spirochaetae (2.3%)	*Spirochaetaceae* (2.2%)
	Lentisphaerae (1.1%)	RFP12 gut group (0.4%) *Victivallaceae* (0.2%) BS5 group (0.2%) WCHB1-25 group (0.1%)

Archaea (0.7%)	Euryarchaeota (100%)	*Methanobacteriaceae* (49.7%) *Methanomassiliicoccaceae* (50.3%)

Eukaryota (21.8%)	SAR group (96.7%)	Intramacronucleata (90.4%)
	Opisthokonta (3.3%)	Neocallimastigomycota (2.7%)

*Only phyla identified with a relative abundance higher than 1% and corresponding families are reported. At the superkingdom level, relative abundances (in parentheses) are expressed as a percentage of total ssu rRNA reads, whereas for phyla and families, these values are reported as a percentage of ssu rRNA reads in the corresponding superkingdom.*

**Table 2:** Quantification and relative abundances of main populations of rumen microorganisms evaluated by qPCR and RNA-sequencing.

	qPCR					RNA-Seq
	DNA					cDNA					ssu RNA/DNA ratio					Relative abundance				
	Copies/μg DNA ± SD	Relative abundance	Copies/μg cDNA ± SD	Relative abundance		
Bacteria (16S) *Bacteria (16S)*	1.20 × 10^10^ ± 3.53 × 10^9^	99.56%	5.35 × 10^10^ ± 2.05 × 10^9^	76.07%	4.46	*77.73%*
Methanogenic archaea (16S) *Euryarcheota (16S)*	2.12 × 10^7^ ± 2.88 × 10^6^	0.18%	1.20 × 10^8^ ± 1.41 × 10^3^	0.17%	5.66	*0.68%*
Protozoa (18S) *Intramacronucleata (18S)*	3.08 × 10^7^± 1.17 × 10^7^	0.25%	1.67 × 10^10^ ± 1.06 × 10^6^	23.74%	542.2	*20.45%*
Neocallimastigomycota (ITS1) *Neocallimastigomycota (18S)*	1.47 × 10^6^ ± 4.63 × 10^5^	0.01%	1.20 × 10^6^ ± 1.34 × 10^1^	0.02%	0.82	*0.61%*

*Relative abundances are given as a percentage of total ssu copies (qPCR) or total ssu reads (RNA-Seq). For qPCR results, each value is the mean (*n* = 6) ± standard deviation. The precise denomination of microbial groups and the gene targeted are indicated in the first column, in italics for RNA-Seq data.*

Recently, Jiang et al. (2016) highlighted that the rumen microbiome lacks sufficient representative reference genomes to perform robust searches of sequence similarities. We hypothesized that sequences originating from the gut of other mammals could extend the annotation of rumen metatranscriptomics data compared with generalized databases, such as the NCBI NR database. We compared BLAST results obtained with NR and two versions of the human gut microbiota gene catalog from the MetaHIT project as reference databases (Qin et al., 2010; Li et al., 2014). BLAST based similarity search using the NR database resulted in 38.8% significant BLAST hits, which were only increased by a further 4.5% when the MetaHIT databases were used, with the best results obtained with MetaHIT V3 (Supplementary Figure S1A). Evaluation of the bitscore of the best hits which reflects the quality of the alignment indicated that the NCBI NR database provided only around 50% of the best bitscores (Supplementary Figure S1B). Thus, available gene catalogs from other mammals provide only a limited enhancement of annotation of sequenced data. Furthermore, taxonomic annotation of sequences from mammalian catalogs is extremely limited and did not allow complete taxonomic binning of putative mRNA in the present work. Consequently, gene catalog specific to rumen is really needed especially for rumen eukaryotes that do not have representatives in current gut gene catalogs. Taxonomic binning of putative mRNA was only picked from BLAST results obtained with the NCBI NR database. Based on a lowest common ancestor (LCA) analysis, putative mRNA were primarily related to bacteria (93.7% of reads with a BLAST hit (e-value < 1e-05), eukaryota (3.0%), archaea (Euryarcheota; 1.5%), and viruses (0.05%). Regarding bacteria, the phyla of Bacteroidetes (37.6%), Firmicutes (38.9%), Spirochaetes (3.6%), Proteobacteria (3.3%), and Fibrobacteres (2.9%) were the most active, with an abundance of more than 1% of putative mRNA. At the family level, *Prevotellaceae* (19.3%), *Lachnospiraceae* (5.3%), *Ruminococcaceae* (5.0%), *Spirochaetaceae* (3.0%), *Bacteroidaceae* (2.7%), *Fibrobacteraceae* (2.8%), and *Clostridiaceae* (1.3%) were among the most active microorganisms. Overall, both approaches based on rRNA and non-rRNA analysis resulted in the same representation of dominant active bacteria in the ruminal community. However, about half of the reads were assigned to taxonomic ranks higher than family using the LCA method. The LCA assignments to high-level taxa are usually associated to conserved sequences among several taxa, and thus could bias the results. In contrast to the analysis of rRNA in which eukaryotes represented a quarter of the active microbiota (**Figure 1**), only a low number of reads attributed to eukaryotes (3.02%) were retrieved. Sequences were mainly associated with rumen protozoa (*Ophryoscolecidae*; 0.70%), Amoebozoa (0.15%), rumen fungi (*Neocallimastigaceae*; 0.11%), and Ascomycota (0.02%), whilst the remaining eukaryotic taxa were discarded due to erroneous identification of sequence sources in the database.

The functional binning of putative mRNA (based on KEGG orthology) indicated that the main functions expressed by the rumen microbiota were primarily related to metabolism (47.6% of assigned reads) and genetic information processing (14.4%). Other functional assignments were linked to bacterial and viral virulence factors associated to human infectious diseases (7.5%), environmental information processing (5.4%), organismal systems (4.9%, mostly plant-pathogen interaction), and cellular processes (4.7%). Within metabolic functions, carbohydrate metabolism was the most important (16.1% of total assigned reads), followed by energy metabolism (10.1%), whereas other metabolic processes accounted for less than 7% of total assigned reads. Metabolic functions, metabolism of carbohydrates in particular, are usually the most expressed by gut microbiota (Gosalbes et al., 2011; Dai et al., 2015; Lee et al., 2015) underlining the importance of this activity in gut ecosystems.

### Overview of CAZymes Expressed by the Rumen Microbiota

Formally, the “CAZyme” term embraces all CAZymes and their associated non-catalytic carbohydrate-binding modules (CBM), involved in the synthesis or degradation of complex carbohydrates (Cantarel et al., 2009). While glycosyltransferase (GT) transcripts were found in the metatranscriptome (3816 reads) of the total rumen content, these were not taken into account since they were related to carbohydrate synthesis and not degradation. Similarly, families of auxiliary activities (AA), mainly represented by lignin degrading enzymes, detected with only 161 reads in total (9 families) were not further analyzed (Supplementary Table S2). In the present analysis, emphasis was placed on the searching for catalytic domains of GH, CE, and PL (further designated as CAZymes in the manuscript) which are key enzymes involved in the deconstruction of polysaccharides. A total of 12,237 sequences of CAZymes, including GH (10,209), CE (1,404), and PL (624) were retrieved, that collectively represented about 1.0% of non-rRNA reads. Based on the LCA analysis, around 41% of non-rRNA reads were related to 89 different genera, and the remaining reads were linked to higher taxonomic levels (Supplementary Table S3). CAZyme reads pertained predominantly to bacteria from the phyla Bacteroidetes (43.8%; *Prevotella* sp., 18.8%; *Bacteroides* sp., 3.5%), Firmicutes (23.4%; *Ruminococcus* sp., 7%; *Clostridium* sp., 0.8%), Fibrobacteres (4.6%; *Fibrobacter* sp., 4.6%), and Spirochetes (0.9%; *Treponema* sp., 0.8%), with lower numbers assigned to eukaryotes (5.4%; *Neocallimastix* sp., 0.3%; *Piromyces* sp., 0.15%; *Epidinium* sp., 0.3%, and *Polyplastron* sp., 0.3%; Supplementary Table S3). Diversity of these CAZymes was relatively high given that 99 GH (out of the 135 listed in the CAZy database), 14 CE (out of 16), and 13 PL (out of 23) families were identified, with a broad range of substrates ranging from complex polysaccharides to oligosaccharides (Supplementary Table S2). Overall, detected CAZymes were dominated by families known to exhibit enzymatic activities for hydrolysis of plant polysaccharides (hemicellulose, cellulose, pectin, and starch; Supplementary Table S2), consistent with isolation of rumen content from a cow fed a diet containing both grass silage and cereals as sources of plant cell walls and starch (Bayat et al., 2015). Major CAZyme transcripts encoded polysaccharidases (GH13, 9, and 10 for example) or oligosaccharidases (GH2 and GH3), the latter often being the most represented in rumen metagenomes (Brulc et al., 2009; Hess et al., 2011). The CAZyme profile reported here differs substantially from two recent reports on the rumen metatranscriptome (Dai et al., 2015; Shinkai et al., 2016). For example, the GH13 family (amylases) was consistently found in the metatranscriptomes of two cows to represent about 20% of the GH (Dai et al., 2015), but only 6% in the present analysis. Similarly, the profile of hemicellulases also differed between the present and previous studies (Shinkai et al., 2016). Several factors may contribute to these apparent discrepancies including animal diet, methodology (RNA extraction), and source of rumen microbiome (sampling site and time), as well as the relative abundance of planktonic and attached communities involved in fiber degradation. Although few variations were observed between the two samples from the same diet in the previous metatranscriptome of Dai et al. (2015), animal variability could also be involved.

Distinct domains can be associated to the catalytic domains mentioned above. Typically, CBM enable to potentiate and optimize enzymatic hydrolysis by maintaining the catalytic domain near the substrate (Abbott and van Bueren, 2014). A total of 1,780 putative CBM were retrieved and assigned to 52 families, with CBM37, CBM50, CBM4, CBM48, and CBM6 families being the most abundant (Supplementary Table S2). While CBM50 is common within bacteria^1^, CBM37 (260 reads in this metatranscriptome) has only been found in *Ruminococcus albus* and thought to facilitate adhesion of the bacterium to the substrate (Ezer et al., 2008; Rakotoarivonina et al., 2009). The detection of putative mRNA containing dockerin and cohesin domains suggests that cellulosomes are employed by rumen microorganisms *in vivo* (Supplementary Table S2). Cellulosomes represent multi-enzymatic machineries that enable the activity of several fibrolytic enzymes to act synergistically (Flint et al., 2012). The detection of *susC* and *susD* homologs also underlines the use of PUL by the rumen microbiota (Supplementary Table S2). These genetic loci have been reported in several rumen metagenomes (Pope et al., 2012) and proposed as a mechanism for polysaccharide deconstruction in the rumen (Naas et al., 2014). In the present metatranscriptomics data, their occurrence was higher than cellulosome associated domains (dockerins and cohesins; Supplementary Table S2).

### Degradation of Plant Polysaccharides in the Rumen of a Cow Fed a Mixed Diet: CAZymes and Microbial Communities Involved

The experimental cow received a mixed diet, similar to commercial conditions, containing 23% of acid detergent fiber (comprising cellulose and lignin) and 17% of hemicellulose, reaching to 40% of fiber, among which 32% was potentially digestible. The diet contained also 12% of starch and 3.3% sugar. In accordance with the diet composition, cellulase and hemicellulase CAZyme families predominated, with 26.5 and 43.2% of CAZyme reads, respectively.

**FIGURE 2:**
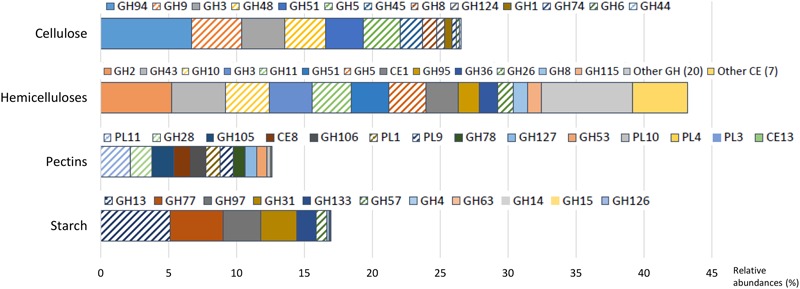
**Relative abundances of transcripts encoding carbohydrate-active enzymes (CAZymes) families with known activities involved in the breakdown of cellulose, hemicelluloses, pectins, and starch.** Families of endo-acting enzymes which may play a pivotal role in these processes are highlighted by hatches. GH, glycoside hydrolase; CE, carbohydrate esterase; PL, polysaccharide lyases.

**FIGURE 3:**
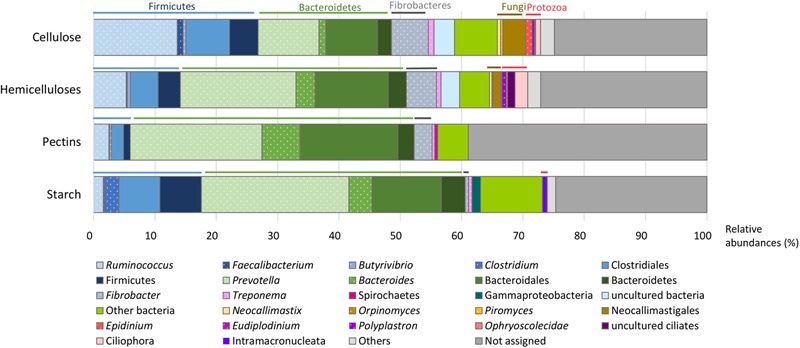
**Putative microorganisms involved in the breakdown of cellulose, hemicelluloses, pectins, and starch.** Taxonomic binning of transcripts encoding CAZymes families with known activities involved in the breakdown for each polysaccharide type was performed using the Lowest Common Ancestor algorithm, leading to a different taxonomic rank (genera are highlighted by dots). Relative abundances were calculated from the total of CAZymes targeting each plant polysaccharide.

Cellulases were represented by 13 families (**Figure 2** and Supplementary Table S2), mainly encoding endoglucanases and cellobiohydrolases, except GH1 and GH3 (in majority β-glucosidases) and GH94 (cellobiose/cellodextrin phosphorylase). The potential origin of endo-acting cellulase transcripts was mostly from the bacterial genera *Ruminococcus* and *Fibrobacter*, fungi (*Neocallimastix* sp., *Piromyces* sp., *Orpinomyces* sp.), and protozoa (*Epidinium* sp., *Polyplastron* sp.) while oligosaccharidases (GH1, GH3, and GH94) originated mainly from *Prevotella* sp. (**Figure 3** and Supplementary Table S3). GH94 is not usually reported as abundant in the rumen metagenome, but was the most expressed family in the present metatranscriptome. Cellobiose phosphorylase activity has been demonstrated for rumen strains of *Ruminococcus, Prevotella*, and *Fibrobacter* (Wells et al., 1995; Lou et al., 1996, 1997). Present results highlight the possibility that phosphorylation could be a common mechanism for the degradation of oligosaccharides released during cellulose hydrolysis. Putative mRNA encoding endo-acting cellulases from GH9, GH48, and GH5 families, that are the most widely studied in rumen microbiology, were among the most abundant in this dataset. Although GH9 and GH5 sequences were over-represented in rumen metagenomic data (Brulc et al., 2009; Hess et al., 2011; Wang et al., 2013), GH48 sequences have been always identified at low frequency. Indeed, genes encoding these families are usually found in high copy numbers in cellulolytic bacterial and fungal genomes, except that GH48 genes are present in monocopies in bacteria^1^. In the present study, expression of GH48-encoding genes was rather high (3.0% of CAZyme transcripts in our sample), consistent with earlier reports (Dai et al., 2015), providing further support for GH48 enzymes playing an essential role in plant cell wall degradation. Surprisingly, GH48 transcripts were not identified in Shinkai et al. (2016) metatranscriptome. Cellulases from GH48 are pivotal enzymes in bacterial (*Clostridium thermocellum, Ruminococcus flavefaciens*) and fungal cellulosomes (Steenbakkers et al., 2002; Bayer et al., 2004). In our data, the majority of GH48 transcripts were from bacteria (89.4%; Supplementary Table S3) and mainly affiliated to *Ruminococcus* (Supplementary Table S3), and to a lesser extent, rumen fungi (Neocallimastigomycota). Interestingly the major cellulase genes expressed by *Fibrobacter* in the present bovine rumen sample encode GH45 enzymes (Supplementary Figure S2). *F. succinogenes* S85 genome contains 4 GH45 genes while other cellulase genes are found in higher numbers^1^ (12 GH5, 9 GH9, and 6 GH8). This suggests that *F. succinogenes* GH45 genes were expressed at high levels in the rumen sample analyzed presently. Only a small number of GH45 genes have been identified in bacteria^1^ and few of them have been characterized (Gilbert et al., 1990; Seon Park et al., 2007). *F. succinogenes* GH45 enzymes merit further investigation given the likelihood of having a major role in fiber degradation by this bacterium *in vivo*.

With respect to hemicellulose hydrolysis, putative mRNA encoding enzymes implicated in the degradation of xylans, xyloglucans, and mannans were retrieved for 32 GH families and 8 CE families (**Figure 2** and Supplementary Table S2). Nonetheless, only 13 families were observed with a relative abundance greater than 1%, accounting for 32.4% of CAZymes. These families include endoxylanases (GH43, GH10, GH11, GH51, GH5, GH26, and GH8), mannanases (GH5 and GH26), oligosaccharidases as well as debranching enzymes (GH2, GH43, GH3, GH51, GH95, GH36, and GH8), and feruloyl and acetyl xylan esterases (CE1). The high relative abundance of transcripts encoding the families GH10 and GH11 (exclusively comprised of endoxylanases) within the CAZyme metatranscriptome underlines their pivotal role in xylan degradation *in vivo*. Transcripts encoding these two families principally originated from protozoa (24.1% of the GH10-GH11 transcripts), *Ruminococcus* sp. (13.8%), *Fibrobacter* sp. (8.0%), and *Prevotella* sp. (7.1%) (Supplementary Table S3). Furthermore, GH26, a family of mannanases, was relatively enriched in the present dataset and mostly related to the same bacterial genera. The high expression of genes encoding GH43 in the present analysis as well as transcriptomic data from pure strains grown on xylan (Dodd et al., 2010; Sawhney et al., 2015) suggested an important role in hemicellulose degradation. Indeed, GH43 family contains enzymes with various specificities including oligosaccharidases and debranching enzymes^2^ (β-xylosidase, α-L-arabinofuranosidase, arabinanase). Debranching enzymes are key components of the enzymatic machinery of hemicellulolytic microorganisms that facilitate the access of endo-acting enzymes to their substrate.

Due to their intimate association with cellulose and hemicellulose, degradation of pectins may accelerate the process of complete plant cell wall deconstruction. Currently, very scarce metatranscriptomic data of pectin degradation are available. Previous rumen metatranscriptomes identified a few PL reads, but the full array of enzymes implicated in pectin degradation was not analyzed in details (Shinkai et al., 2016; Dai et al., 2015). Conversely, 14 CAZymes (6 GH families, 6 PL families and 2 CE) involved specifically in pectin degradation were identified in this dataset, representing 12.6% of total CAZyme reads. Putative mRNA encoding endo- and exo-acting enzymes from the families PL11 (2.2% of CAZymes), GH28 (1.6%), PL1, and PL9 (∼1.0%), and debranching enzymes from the family CE8 (1.2%) were the most abundant (**Figure 2** and Supplementary Table S2). They have been exclusively assigned to bacteria, mainly to Bacteroidetes (46.3%; *Prevotella* sp., 21.5%; *Bacteroides* sp., 6.2%*)*, and the *Ruminococcus* (7.4%) and *Fibrobacter* (6.6%) genera (**Figure 3** and Supplementary Table S3). Strains of these genera can degrade and utilize pectin sugars, except for *Fibrobacter succinogenes* that cannot utilize products of pectin hydrolysis (Gradel and Dehority, 1972; Osborne and Dehority, 1989; Marounek and Duskova, 1999; Dongowski et al., 2000). The substantial proportion of pectinase transcripts in our dataset may be due to the presence of pectins in the diet of the animal (less than 3%), but also to cotranscription of pectinase and cellulase/hemicellulase genes by rumen microorganisms, as shown previously for *Ruminococcus flavefaciens* by transcriptome analysis (Berg Miller et al., 2009). Polygalacturonase and pectate lyase genes have been identified by metagenomics analysis of the rumen microbial community in a sheep (Yuan et al., 2012), in which pectinase genes were affiliated to *Butyrivibrio, Prevotella, Bacteroides*, and *Fibrobacter* consistent with the present study, other than very few reads close to *Butyrivibrio* sequences were detected. It appears that pectinolytic activity is mainly associated to the bacterial genera known to be active on the main plant cell wall polysaccharides, i.e., cellulose and hemicelluloses. Nonetheless, certainly due to limitations in databases, a third of the transcripts associated to pectin degradation were not taxonomically affiliated in the present analysis (**Figure 3** and Supplementary Table S3), raising the prospect that other taxa comprising rumen fungi (Kopečný and Hodrová, 1995) may also be involved in ruminal hydrolysis of pectin polymers.

Similar to other plant polysaccharides, total hydrolysis of starch requires the concerted action of several enzymes comprising debranching enzymes, endo- and exo-amylases. In the present study, with a diet containing 12% of starch, transcripts of amylolytic enzymes were found in 11 GH families (**Figure 2** and Supplementary Table S2) and accounted for 16.9% of CAZyme reads. They were related to Bacteroidetes (43.0%; *Prevotella*, 24.1%; *Bacteroides*, 3.8%), Firmicutes (17.6%; *Clostridium*, 2.4%; *Ruminococcus*, 1.6%), and Gammaproteobacteria (1.4%) (**Figure 3** and Supplementary Table S3). The main cultivated amylolytic rumen strains belong to *Prevotella* and *Butyrivibrio* species, *Streptococcus bovis*, and *Selenomonas ruminantium* (Fonty and Chaucheyras-Durand, 2007). These results demonstrate that *Prevotella* is the major player in the studied cow rumen, but *Bacteroides* and *Clostridium* may also be significant amylolytic genera, although very few, if any, rumen isolates from these genera have been characterized. Protozoa function as amylolytic microorganisms in the rumen (Williams and Coleman, 1997), and with a diet containing 12% starch we expected to find more transcripts affiliated to protozoal amylases. Nonetheless, they were detected at only a low relative abundance (GH13; 0.1%). Only 2 protozoal GH13 are available in the NR database. Given that identification of reads depends largely on sequences available in databases, a high proportion of protozoal amylase sequences are most probably not retrieved and remain unassigned.

### Contribution of Eukaryotes to Fibrolytic Processes in the Rumen

**FIGURE 4:**
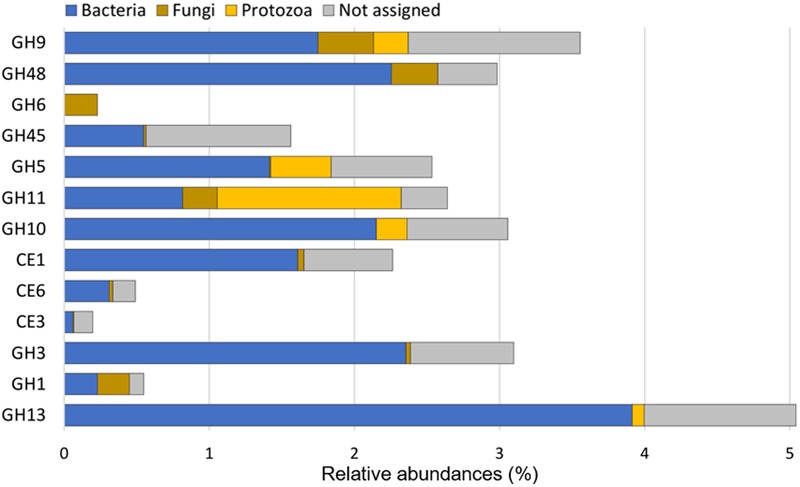
**Relative contribution of bacteria, protozoa, and fungi to putative mRNA encoding CAZyme families for which sequences of eukaryotes are available.** Abundances were calculated relative to the total CAZyme (GH+CE+PL) reads. These data indicate that eukaryotes, protozoa in particular, may contribute substantially to hemicellulose and cellulose degradation in the rumen.

The contribution of eukaryotes to ruminal fiber degradation *in vivo* has not been fully elucidated. Metatranscriptomics is therefore a relevant approach to address this knowledge gap. CAZyme transcripts potentially originating from eukaryotes have been investigated (Qi et al., 2011), but the data generated did not permit to evaluate the abundance of transcripts from eukaryotes relative to bacteria. Shinkai et al. (2016) did not detect eukaryotic transcripts during the analysis of a cow rumen sample. In contrast, Dai et al. (2015) reported that eukaryotes can contribute to 18% of cellulase (GH5, GH6, GH9, GH44, GH45, and GH48) and 3.5% of hemicellulase (GH8, GH10, GH11, GH26, GH28, GH51, GH53, GH67, and GH78) transcripts. Nevertheless, about 20% and 40% of cellulase and hemicellulase-encoding transcripts with as low as 30 to 60% of identity with known sequences were included, raising the likelihood of false positives. In the present study, CAZyme transcripts from eukaryotic origin and encoding the same GH families represented 20.5 and 15.2%, respectively, with at least 60% identity with a known sequence. Interrogation of CAZyme families for which eukaryotic sequences were available (**Figure 4**), indicated that fungal sequences contributed up to 10% to cellulase (GH9 and GH48) and hemicellulase (GH11) families. The GH6 family appeared to be specific to fungi. The most notable eukaryotic contribution was detected for GH11, with protozoal reads representing up to 48% of total GH11 transcripts. Protozoa also contributed significantly to cellulase and hemicellulase sequences from the GH5, GH9 and GH10 families. Taking into account the low number of eukaryotic CAZyme sequences in databases^3^ and the proportion of protozoa and fungi in the rumen microbial population (around 0.00001%), the abundance of putative transcripts encoding CAZyme families from eukaryotic origin supports the hypothesis that, under our experimental conditions, eukaryotes contributed significantly to ruminal fiber degradation. Such a contribution is probably underestimated as many of the protozoal and fungal CAZyme sequences are currently unavailable. The role of protozoa in fibrolysis has long been controversial, as they have been primarily considered as very active protein and starch degraders (Williams and Coleman, 1997). The very high rRNA/rDNA ratio observed for protozoa (**Table 2**), suggesting their high activity, recommends that protozoa function and ecology should deserve further investigations.

### Evidence for the Contribution of Uncharacterized and Unknown Bacterial Communities to Fibrolytic Processes in the Rumen

Using a bitscore threshold of 90 for the LCA analysis, the present analysis concentrated on sequences that shared the highest identity with known references to characterize with a high degree of certainty the taxonomic contribution of the rumen fibrolytic community. Using this approach, it was possible to confirm the important role of well-studied rumen bacteria to degradation of the fibers included in our experimental diet (i.e., *Prevotella* sp., 18.7% of fiber degrading CAZymes; *Ruminococcus* sp., 5.6%, and *Fibrobacter* sp., 4.0%). In general the CAZyme expression profile correlated well with the ability to degrade cellulose, hemicellulose, pectin or starch *in vitro* (Supplementary Figure S2) (Béra-Maillet et al., 2000; Berg Miller et al., 2009; Dodd et al., 2010; Youssef et al., 2013; Burnet et al., 2015; Couger et al., 2015). Other bacteria may well participate, given that sequences of *Bacteroides* sp. (3.4%), *Clostridium* sp. (0.8%), *Treponema* sp. (0.5%), and *Butyrivibrio* sp. (0.4%) were also identified (Supplementary Table S3). While these genera are frequently retrieved from rumen metagenomes (Patel et al., 2014), few ruminal isolates are available for biochemical characterization. A third of the sequences retrieved in the present rumen metatranscriptome may originate from more distantly related strains or unknown microorganisms, since 26% of reads with as low as 30% of identity with a known sequence were not assigned using MEGAN (bitscore < 90) and 3% did not result in a significant BLAST hit (e-value > 1e-05) (Supplementary Table S3). Generation and availability of more genomes is required to advance knowledge on the rumen fibrolytic community. For this reason, the use of draft genomes reconstructed from a metagenome of switchgrass-attached bacteria (Hess et al., 2011) that may well include fibrolytic bacteria was also investigated. Using only 15 genomes, 1.7% of the CAZyme reads were retrieved with an identity higher than 90% (Supplementary Table S4). Genomes that resulted in improvement of similarity search were related mainly to Bacteroidales (72.1%) and Clostridiales (20.6%) (Supplementary Table S4).

## Conclusion

Even though shot-gun metagenomics approaches have been successfully applied in the past to decipher the fibrolytic potential of the rumen microbiota, metatranscriptomics approaches offer the opportunity to investigate the relative contribution of metabolically active members of the rumen microbial community. Currently, only two metatranscriptomes focusing on the rumen fibrolytic communities have been published (Dai et al., 2015; Shinkai et al., 2016). Using different experimental conditions (mixed diet, sampling before morning feeding), the present study completes the available snapshots of the key features of ruminal fiber degradation by confirming the main contributors to this function, and generating new information highlighting the role of eukaryotic microorganisms.

Despite a diversified CAZyme repertoire harbored by the genome of rumen fibrolytic microorganisms, few enzyme families were highly expressed *in vivo*. Their high relative abundance suggests an active contribution to the fibrolytic process in the animal studied. Present data highlighted pivotal families involved in the *in vivo* deconstruction of cellulose (GH9, GH48, GH5, and GH94), hemicellulose (GH10, GH11, and GH43), pectin (PL11 and GH28), and starch (GH13). Illustrating the strength of metatranscriptomic approaches, GH families which are typically near-absent in metagenomics data such as GH48 and GH94 were among the most represented in CAZyme transcripts within our dataset. However, it should be pinpointed that transcripts quantification does not always reflect exactly the enzymatic activities produced as many post-transcriptionnal regulations can occur in the microorganisms. Otherwise it is highly probable that the CAZyme profile is dependent on the relative amounts of forage and concentrate, and the type of plant polysaccharides in the host ruminant diet. Also, the profile of the CAZyme transcripts may evolve during digestion of the meal and the colonization of ingested feed particles. Thus, the CAZyme expression picture outlined here must be considered as a snapshot of the fibrolytic community of one cow at a single time point. Future studies should expand the present approach to give a more complete picture of fiber degradation in the rumen, for example by monitoring CAZyme transcripts throughout the digestion process, or by comparing the degradation activity of different management systems.

As found by Dai et al. (2015), bacteria from the most studied genera of rumen fibrolytic strains (*Prevotella, Ruminoccocus, Fibrobacter*) were found to be among the most active in the studied cow rumen, underlying that previous culture-based analyses have certainly provided a valuable overview of the major bacterial actors in the rumen. The main originality of the present study is to use with great care metatranscriptomics data to assess the relative contribution of eukaryotes to polysaccharide breakdown, highlighting the fact that this approach could overcome many of the current limitations of *in vitro* and *in vivo* studies. Eukaryotes, and especially protozoa, were found to contribute significantly to the expression of the CAZyme families GH9, GH10, GH48, GH5, and GH11 despite a low abundance in the rumen and the limited number of reference sequences in available databases. These findings suggest that the metabolic activity of protozoa, and maybe also fungi, is more important in cellulose and hemicellulose degradation than previously thought, a contribution likely to be underestimated due to the lack of gene catalogs. These results may motivate further exploration of the role and the functions of protozoa in the rumen.

Overall the complexity of ecology and function of the rumen microbiota leads to huge challenges in acquiring sequence data. The lack of sequenced genomes still represents a tremendous barrier to identify genes, especially CAZymes, and many of them remained as putative in our study. We assessed opportunities to circumvent analytical limitations (use of the human gut metagenome catalog and draft genomes reconstructed from metagenomic data). Unfortunately, enhancement of sequence annotation was very limited. Completion of the rumen microbiome catalog as well as better annotation of genomes is really fundamental to better understand the rumen microbiota.

## Materials and Methods

### Animal Experiment, Diet, and Sampling

All experimental procedures were approved by the National Ethics Committee (Hämeenlinna, Finland) in accordance with the guidelines established by the European Community Council Directive 86/609/EEC (European Union, 1986). The analyzed rumen sample was obtained from a lactating dairy cow fed with a total mixed ration (forage:concentrate ratio 50:50, on a dry matter content) based on grass silage. Details of the diet fed have been reported previously (Bayat et al., 2015). In brief, the total mixed ration contained (g/kg dry matter) neutral detergent fiber (401), acid detergent fiber (228), and starch (120). Samples were taken before the morning feeding from five different sites in the rumen, composited, and mixed thoroughly to obtain a representative sample of rumen contents. For molecular analysis, rumen content was subsampled (50 g) and mixed with 100 mL of RNAlater (Thermo Fisher Scientific, Waltham, MA, USA) to prevent RNA degradation. The mixture was maintained overnight at +4°C and then stored at –80°C until nucleic acid extraction.

### DNA and RNA Extraction from Rumen Sample

DNA and RNA were extracted from the RNAlater-preserved sample following thorough mixing after thawing. DNA isolation was performed in triplicate from 250 mg of centrifuged rumen content according to Bayat et al. (2015). DNA samples were assessed for purity and quantity using a NanoDrop 1000 spectrophotometer (Thermo Fisher Scientific, Waltham, MA, USA), and stored at –20°C until molecular analysis. Total RNA was extracted in triplicate from 400 mg of centrifuged rumen content using Trizol (Thermo Fisher Scientific, Waltham, MA, USA). In brief, samples were homogenized with ∼100 mg of 0.1 mm zirconia beads (BioSpec Products, Bartlesville, OK, USA) and 1.2 mL of Trizol using the Fast Prep-24 instrument (MP Biomedicals, Irvine, CA, USA). All subsequent steps were in accordance with the recommendations from the manufacturer. RNA precipitation was performed using a mix of isopropanol and a saline solution (NaCl 1.2 M, disodium citrate 0.8 M) as reported by Béra-Maillet et al. (2009). Total RNA was subjected to DNase treatment (Nucleospin rDNase set, Macherey-Nagel, Düren, Germany). The integrity of RNA was assessed with the Agilent 2100 Bioanalyzer using the RNA Nano Chip (Agilent Technologies, Santa Clara, CA, USA). Quantity of RNA was assessed with the Nanodrop 1000 spectrophotometer (Thermo Fisher Scientific, Waltham, MA, USA). Triplicates of total RNA were pooled and the resulting RNA solution (with a RIN of 9.4 and a RNA ratio of 2.0) was stored at –80°C until further processing.

### Reverse-Transcription and Quantification of Microbial Populations

Abundances of the main groups of rumen microorganisms were determined by quantitative real-time PCR (qPCR) from both DNA and RNA. RNA was reverse-transcribed into cDNA with 100 ng of starting material and the Superscript II Reverse transcriptase kit (Invitrogen, Thermo Fisher Scientific, Waltham, MA, USA) using random primers and following the manufacturer’s instructions. It was considered that the reaction resulted in the production of 100 ng of cDNA. Q-PCR were performed in duplicate with the three technical replicates of extraction using 40 ng of DNA and 1 ng of cDNA templates (Bayat et al., 2015). Bacteria and methanogenic archaea were quantified by targeting the 16S rRNA gene, and rumen ciliate protozoa based on amplicons of the 18S rRNA gene (Supplementary Table S9). For Neocallimastigomycota, the ITS1 region was targeted to quantify rumen specific fungi. ITS regions are widely used as phylogenetic markers for fungal community analysis and their half-life in the pre-rRNA is sufficient to allow quantification (Lindahl et al., 2013). A reverse transcriptase negative control was included to ensure the absence of genomic DNA contamination. Standards were used to determine the absolute abundance of microbial groups, expressed as the number of DNA copies per μg of DNA or the number of cDNA copies per μg of cDNA. Efficiency of the qPCR for each target varied between 97 and 102% with a slope from -3.0 to -3.4, and a regression coefficient above 0.95 in accordance with the MIQE guidelines (Bustin et al., 2009). Relative abundances of ssu (or ITS1) copies of each microbial group was calculated relatively to the total ssu and ITS1 copies of all microbial groups quantified by qPCR. These abundances were determined for each technical replicate and the standard deviation was calculated on the six replicates.

### Messenger RNA Enrichment by rRNA Capture

As no commercial kit dedicated to mRNA enrichment of both the targeted eukaryotic and prokaryotic rRNAs from complex digestive environments is available, an in-house procedure was developed to deplete rRNA from total RNA to analyze the rumen metatranscriptome. This method was based on the specific capture of ribosomal RNAs. In total, 18 capture probe sets targeting approximately 23,000 sequences (16S, 18S, 23S, and 28S rRNA) belonging to 204 rumen genera (bacteria, archaea, protozoa, fungi) were designed from iterative multiple alignments (see Supplementary Materials). Enrichment was performed with a starting material of 10 μg of total RNA (see Supplementary Materials for a detailed protocol). One aliquot of the total RNA sample was used as a control for RNA degradation by completing all the steps without rRNA capture. The other aliquot was treated with a commercial kit (MicrobExpress, Ambion, Thermo Fisher Scientific, Waltham, MA, USA) according to the recommendations of the manufacturer in order to compare the efficiency of both methods (see Supplementary Materials). mRNA enriched fractions obtained with the two methods of rRNA removal were resuspended in the same volume (10 μL). Quality of the control RNA and effective removal of rRNA were controlled using the Agilent 2100 Bioanalyzer and the RNA Nano Chip (Agilent Technologies, Santa Clara, CA, USA), and one tenth of each mRNA enriched fractions.

### Metatranscriptome Sequencing, Sequence Pre-processing and Contig Assembly

Sequencing of total RNA and mRNA-enriched RNA was conducted with an Illumina MiSeq instrument (2 × 300 bp) at the Macrogen Company (Seoul, Korea). Adapter sequences and low quality sequences (*Q* < 30) were trimmed. After quality trimming, a total of 20,746,664 and 18,145,720 reads were obtained for total RNA and mRNA-enriched RNA samples, respectively (Supplementary Table S8). Paired-end reads were merged using the Fastq-join program ^4^ with default parameters. Ribosomal RNA (5S, 5.8S, 16S, 18S, 23S, and 28S) were identified from merged reads using SortMeRNA 2.0 (Kopylova et al., 2012) (**RRID**:SCR_014402) and picked from RNA-seq data. Non-rRNA sequences, theoretically corresponding to putative mRNA, were assembled *de novo* using Trinity (**RRID**:SCR_013048) (Grabherr et al., 2011) with the default parameters. Sequence assembly led to 12,240 and 23,150 assembled transcripts for total RNA and mRNA-enriched RNA, with an average length of 354 and 357 nt, respectively (Supplementary Table S8). Assembly did not result in large contigs, and were therefore uninformative. For this reason, unassembled merged reads were used for further analysis.

### Taxonomic Profiling of the Rumen Microbial Community Based on ssu rRNA Reads

16S (2,421,511 reads) and 18S rRNA (945,524 reads) sequences from the total RNA sample were used to assess rumen prokaryotic and eukaryotic diversity, respectively, using Mothur (**RRID**:SCR_011947; Schloss et al., 2009) following the analysis pipeline used by Li et al. (2016), except that the SILVA database was used as reference database. Briefly, the V1-V3 region of the bacterial sequences from the 50,000 columns long-aligned SILVA reference database was used for bacteria classification. Starting (1046) and ending (13125) positions of the targeted regions regarding Li et al. (2016) was modified according to the use of different databases. The V6-V8 region (position 955–1510) of archaeal sequences of the aligned RIM-DB database (Seedorf et al., 2014) was used for archaea. For the taxonomic analysis of eukaryotes, the regions V3–V4 targeted by Kittelmann et al. (2013) were used. Read count was normalized in FPKM (Fragments Per Kilobase Of Exon Per Million; Mortazavi et al., 2008) to take into account the length differences between the 16S and the 18S rRNA genes. Relative abundances were calculated as a proportion of total ssu RNA reads normalized.

### Taxonomic Assignment and Functional Classification of Putative mRNA Reads

The DIAMOND program (Buchfink et al., 2014) is about 2,500 times faster than the BLAST algorithm (Altschul et al., 1990; **RRID**:SCR_001653) and was used to evaluate the potential for using human gut gene catalog to annotate putative mRNA. Sequence similarities searches with enabled “sensitive mode” and “BLASTX method” were performed with three databases, NCBI non-redundant protein (NR) (**RRID**:SCR_003257), MetaHIT V1 (Qin et al., 2010), and MetaHIT V3 (Li et al., 2014). Taking into account the length of each database, hits with an e-value higher than 1e-05 for NR, 2.5e-07 for MetaHIT V1 and 7.8e-07 for MetaHIT V3 were discarded. In return for its calculation time, DIAMOND was less sensitive than BLASTX and found to omit 8% of the total hits. For the taxonomic assignment of putative mRNA, non-rRNA reads were submitted to sequence similarity searches against the NR database (NR) using BLASTX. Hits with an e-value higher than 1e-05 were discarded. BLASTX results (25 hits for each read) were then used in the MEGAN software version 6 (**RRID**:SCR_011942) (Huson et al., 2011) to determine the taxonomic distribution of putative mRNA reads. The taxonomic level was determined using the lowest common ancestor-based algorithm (LCA) implemented in the software. LCA parameters “Top percent” and “Min support percent” were set to 10 and 1e-05, respectively. The “Min score” parameter was set to 50 as recommended by Huson et al. (2011). Functional classification of putative mRNA based on KEGG (Kyoto Encyclopedia of Genes and Genomes) pathways (Kanehisa and Goto, 2000) was picked from the best BLAST hit obtained among the three databases using the MEGAN software.

### Identification of Transcripts Encoding Carbohydrate-Degrading Enzymes and Determination of Putative Microorganisms Involved in Plant Polysaccharide Degradation

Translated non-rRNA sequences from the mRNA-enriched RNA sample were submitted to a local version of dbCAN (**RRID**:SCR_013208) (Yin et al., 2012) to detect and annotate sequences for Carbohydrate-Active enZymes (CAZymes) (e-value < 1e-05). Dockerin and cohesin domains, and PUL genes (*susC*-like and *susD*-like genes) were predicted based on pfam domains. dbCAN-positive sequences were submitted to BLASTX analysis against a database containing all GH, CE, and PL protein sequences extracted from CAZy (**RRID**:SCR_012909) on July 2014. The local database was implemented using the tool “Extract sequences” of the website www.ahv.dk/index.php BLASTX were also performed against the NCBI NR database. The best BLAST hit obtained with each database is reported in Supplementary Table S3. Due to better matching with reads, BLAST results (25 best hits for each read) obtained with the NCBI NR database were used for taxonomic mining of putative CAZyme transcripts using the MEGAN software as described in the previous section, except that the “Min score” parameter was set to 50 for the Results section “Overview of the CAZymes expressed by the rumen microbiota” to highlight the relatedness of reads with NR database sequences and to 90 for subsequent result sections to ensure a stringent taxonomic affiliation of reads (to target known sequences). dbCAN-positive sequences were further analyzed by searching similarities (BLASTX) with 15 draft genomes of uncultured bacteria adherent to switchgrass from Hess et al. (2011). Genome bins were downloaded from http://portal.nersc.gov/project/jgimg/CowRumenRawData/submission/ Improvement of BLAST results regarding the NCBI-NR database was considered reliable when the identity percent was higher than for the NCBI-NR database for the same alignment length or better.

### Sequencing Data Accession Number

Raw sequence data have been deposited in the NCBI Sequence Read Archive (**RRID**:SCR_001370) under accession number SRP070140.

## Author Contributions

SC-M, FC-D, PM, EP, AB, KS, PP, and EF conceived and designed the experiments. KS and AB performed the animal experiment. SC-M and PL performed all the molecular analysis. SC-M and NP performed bioinformatics analysis. SC-M and EF performed data analysis and wrote the manuscript. NP, PL, FC-D, PM, EP, AB, KS, and PP critically reviewed the analysis of experimental data and the contents of the manuscript. All authors have read and approved the final manuscript.

## Conflict of Interest Statement

The authors declare that the research was conducted in the absence of any commercial or financial relationships that could be construed as a potential conflict of interest.

## Footnotes

1 www.cazy.org

2 www.cazy.org

3 http://www.rmgnetwork.org/hungate1000.html

4 code.google.com/p/ea-utils
