# Supplementary material for: Metatranscriptomics Reveals the Active Bacterial and Eukaryotic Fibrolytic Communities in the Rumen of Dairy Cow Fed a Mixed Diet
Source: Front Microbiol. 2017 Jan 31;8:67. doi: 10.3389/fmicb.2017.00067 (PMC5281551; doi:10.3389/fmicb.2017.00067)
Supplement: Supplementary file 1 [file Data_Sheet_1.pdf]

Supplementary Material

Metatranscriptomics reveals the active bacterial and eukaryotic fibrolytic communities in the rumen of dairy cow fed a mixed diet

Sophie Comtet-Marre[1], Nicolas Parisot[2,$], Pascale Lepercq[1,#], Frédérique Chaucheyras-Durand[1,3], Pascale Mosoni[1], Eric Peyretaillade[2], Ali R. Bayat[4], Kevin J. Shingfield[4,5,†], Pierre Peyret[2] and Evelyne Forano[1,*]

[1]UR454 Unité de Microbiologie, INRA, Saint-Genès-Champanelle, France

[2] EA4678 CIDAM, Clermont Université, Université d'Auvergne, Clermont-Ferrand, France

[3]Lallemand Animal Nutrition, Blagnac, France

[4]Nutritional Physiology, Green Technology, Natural Resources Institute Finland (Luke), Jokioinen, Finland

[5]Institute of Biological, Environmental and Rural Sciences, Aberystwyth University, Aberystwyth, United Kingdom

[$]present address: UMR203 BF2I, Univ Lyon, INSA-Lyon, INRA, Villeurbanne, France

#present address: LISBP-INSA Toulouse, Toulouse, France

[†]Deceased 11 September 2016.

*** Correspondence:** Evelyne Forano: evelyne.forano@inra.fr

Supplementary Methods

Messenger RNA enrichment by rRNA capture. Our in house-procedure is based on the widely used rRNA subtractive hybridization principle. In total , 18 capture probes targeting the large (LSU) and the small (ssu) subunit rRNA of all rumen microorganisms (bacteria, archaea, protozoa, fungi) identified at the genus level were designed (Table S5). Ribosomal RNA sequences of good quality were extracted from the SILVA database (RRID:SCR_006423) (Pruesse et al., 2007). The generated rRNA database comprised about 23,000 sequences belonging to 204 rumen genera (Table S6). For each set of sequences (16S, 18S, 23S, 28S), iterative multiple alignments of rRNA sequences were performed in order to highlight conserved regions. Sequences from the same genus were aligned through MEGA 6 (RRID:SCR_000667) (Tamura et al., 2013) using ClustalW (RRID:SCR_008620) (Larkin et al., 2007) and each alignment was manually curated. Alignments of genera were then aligned successively step by step, considering the phylogenetic position of each genus. In this way, 9 master alignments were obtained (one for each target except for bacterial 16S rDNA resulting in two master alignments). Probes were determined in conserved regions using the HiSpOD

software (RRID:SCR_014403) (Dugat-Bony et al., 2011). Default parameters were used, except for probe length, max similarity of the probe and the non-target sequences (% identity) which were fixed to 24 or 25 nt and 75%, respectively. Probes presenting least potential cross-hybridizations with any coding sequences (CDS) and none with CDS from rumen microorganisms were selected. Capture probes were designed in different regions of the rDNA in order to enhance enrichment effectiveness. Enrichment was performed with a starting material of 10 µg of total RNA. Only DEPC-treated solutions and RNase-free material were used. Solutions were those recommended for the Dynabeads Oligo(dT)25 kit (Invitrogen, Thermo Fisher Scientific, Waltham, MA, USA). In the first step, polyadenylated RNA (eukaryotic and some prokaryotic mRNA) were isolated by capture using Dynabeads Oligo(dT)25 following the manufacturer instructions and kept on ice until the end of the procedure. Capture probes polyadenylated at 5’ position were added at different concentrations (Table S5) to polyA(-) RNA to allow probes to hybridize to their targets during 20 min at 37°C. Probe-rRNA complexes were captured with Dynabeads Oligo(dT)25 as for polyA(+) RNA. This step was repeated three times. Finally, polyA(+) RNA and prokaryotic mRNA-enriched RNA were pooled and ethanol precipitated. One aliquot of the total RNA sample was also used as a control for RNA degradation by following all the steps without rRNA capture (control RNA). Another aliquot of the same sample was treated using a commercial kit (MicrobExpress, Ambion, Thermo Fisher Scientific, Waltham, MA, USA) according to the recommendations of the manufacturer. Quality of the control RNA and effective removal of rRNA were controlled using the Agilent 2100 Bioanalyzer using RNA Nano Chip (Agilent Technologies, Santa Clara, CA, USA).

The efficiency of the enrichment method was assessed both qualitatively and quantitatively, and compared qualitatively to a commercial kit (MicrobExpress, Ambion, Life Technologies SAS, Saint Aubin, France) specifically designed for the removal of bacterial rRNAs (Figure S3). Our in-house capture method enabled the removal of more than 80% of bacterial and protozoal ssu rRNA and about 33 and 54 % of archaeal and fungal rRNA, respectively to be removed (Table S7). The method was less effective for archaeal and fungal rRNA, possibly due to the designed probes not covering all the diversity of these organisms in the rumen. Nonetheless, mRNA enrichment allowed a doubling of the number of available mRNA reads in the present RNA-seq data (Table S8), without introducing any bias (Pearson’s correlation=0.96) (Figure S4). Therefore, the mRNA-enriched RNA sample was retained for functional analysis.

Supplementary Figures

A.

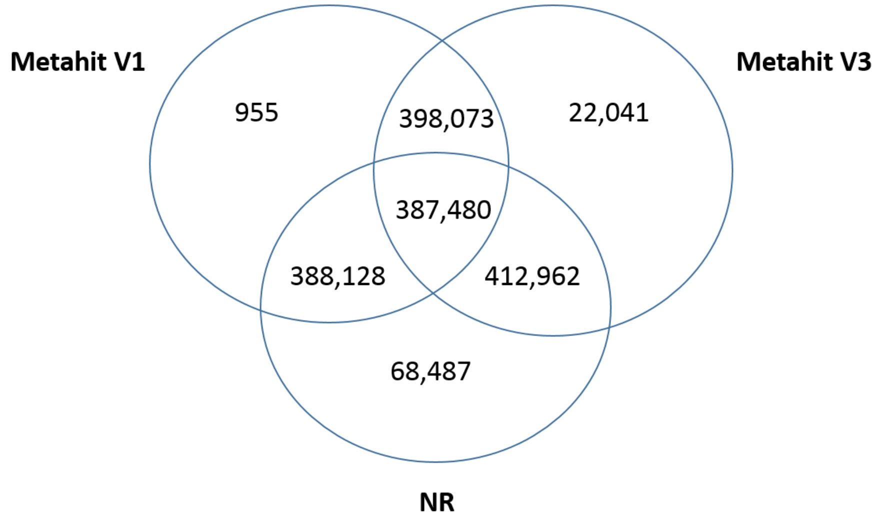

B.

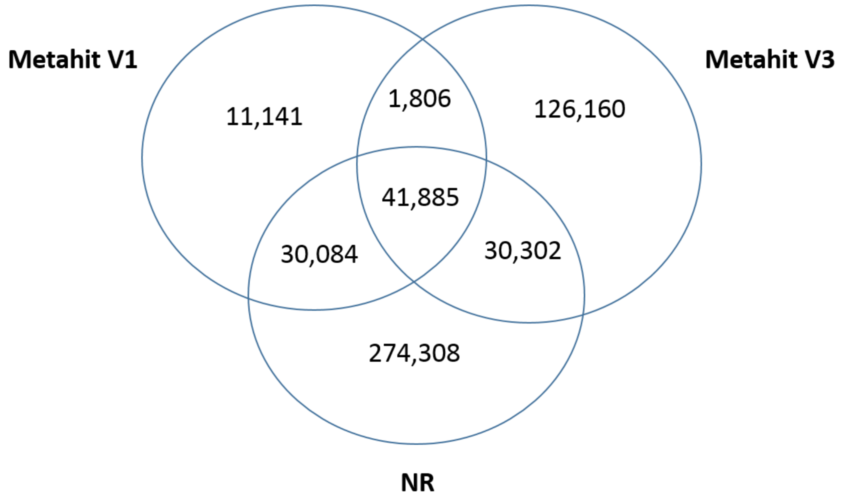

Figure S1. Evaluation of using three databases (NCBI non-redundant protein (NR), Metahit V1, Metahit V3) to annotate the present rumen metatranscriptome dataset.

A. Amount of reads giving a BLAST hit with each of the three databases. Overlaps between circles indicate the amount of reads that have led to a match with a sequence from 2 or 3 databases. Non-overlapping zones indicate the amount of reads matching with only one database.
B. Amount of reads giving the best bitscore within each database. Overlaps between circles indicate the amount of reads which have led to a match with a sequence from 2 or 3 databases.

Prevotella

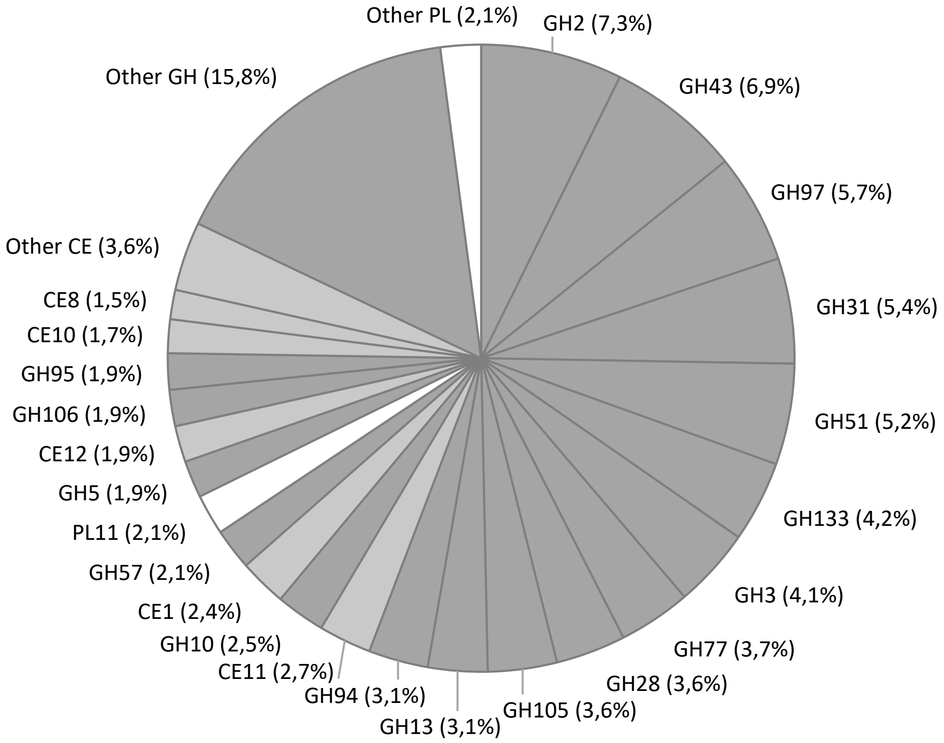

Ruminococcus

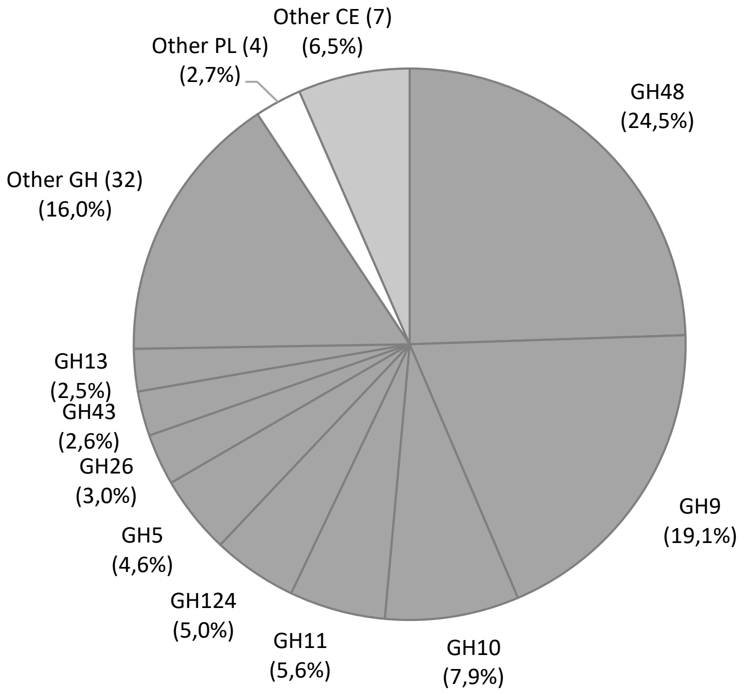

Figure S2. Distribution of glycoside hydrolase (GH), carbohydrate esterases (CE), and polysaccharide lyases (PL) transcripts assigned to the main bacterial genera identified (*Prevotella*, *Ruminococcus*, *Fibrobacter*) using the lowest common ancestor method (bitscore>90). The number of families gathered in “other” is indicated in parentheses.

Fibrobacter

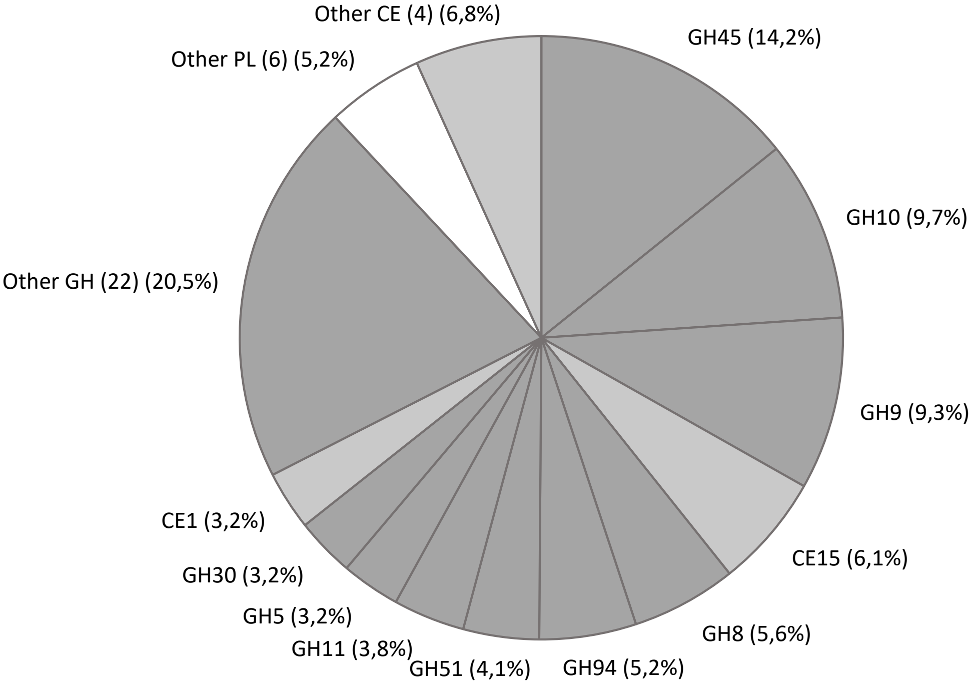

Figure S2 (continued). Distribution of glycoside hydrolase (GH), carbohydrate esterases (CE), and polysaccharide lyases (PL) transcripts assigned to the main bacterial genera identified (*Prevotella*, *Ruminococcus*, *Fibrobacter*) using the lowest common ancestor method (bitscore>90). The number of families gathered in “other” is indicated in parentheses.

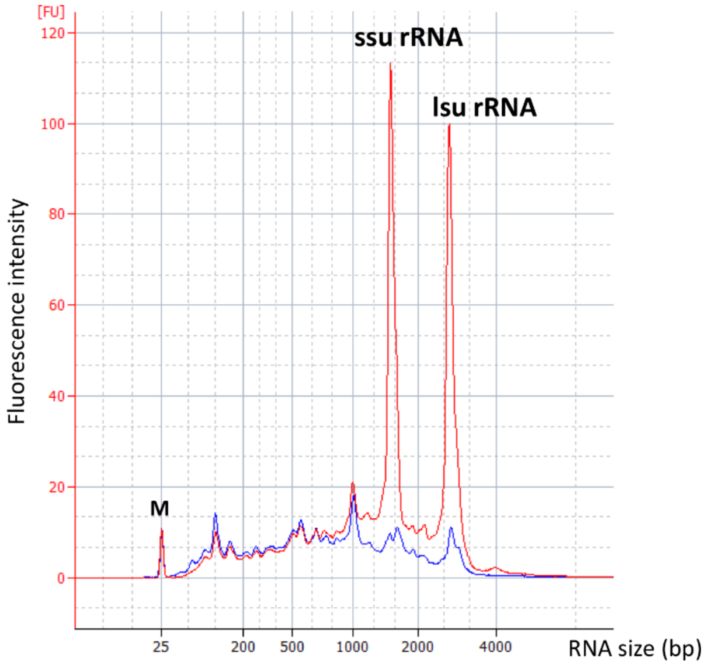

Figure S3. Efficiency of mRNA enrichment methods. Electrophoregrams of total RNA treated with the MicrobExpress kit (Ambion, Life Technologies SAS, Saint Aubin, France) (red) or using the in-house method (blue) that indicate improved performance of the later for removing rRNA peaks (ssu rRNA, lsu rRNA). M: internal marker.

Enrichment was performed with the starting material of 10 µg of total RNA for the 2 methods. Resulting mRNA-enriched fractions were resuspended in the same volume and one tenth was used for quality control.

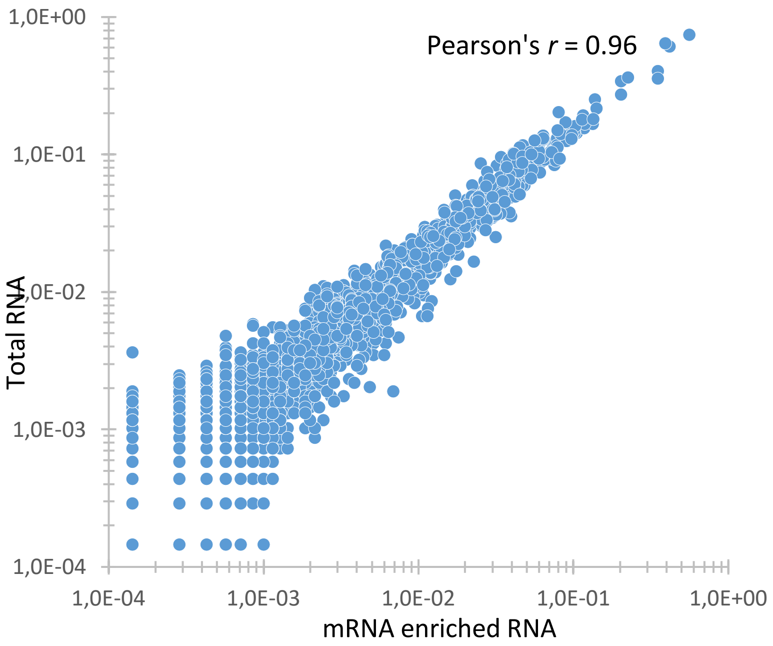

Figure S4. Correlation between relative abundances of KEGG Orthology groups observed with the total RNA and the in house mRNA-enriched method. Pearson's correlation r = 0.96.

Supplementary Tables

Table S1. Relative abundances of rumen microorganisms at the family level based on ssu rRNA analysis.

Excel file Data sheet 2

Table S2. Glycoside hydrolase (GH), carbohydrate esterase (CE), polysaccharide lyase (PL), auxiliary activities (AA), and carbohydrate binding module (CBM) families identified in non-rRNA reads using the dbCAN software (e-value<1e-05). Substrate, known activities, number of reads corresponding to each family, and their relative abundance are indicated.

Excel file Data sheet 3

Table S3. Summary of BLASTX results obtained with the non-rRNA reads annotated as CAZyme by the dbCAN software and their taxonomic binning using the MEGAN software. BLASTX comparisons were performed against the CAZy database (no e-value threshold) and the non-redundant protein database (e-value<1e-05). Only best BLAST hits are reported.

Excel file Data sheet 4

Table S4. Improvement of BLAST results of reads detected as CAZymes by dbCAN, using draft genomes of switchgrass adherent bacteria from Hess *et al.* (2011)

Genome bin	Phylogenetic order	Number of better hits compared with NCBI-NR	Average gain in identity percentage (%)	Minimum gain in identity percentage (%)	Maximum gain in identity percentage (%)
AC2a	Bacteroidales	145	13.38	0.31	43.89
AH	Bacteroidales	101	12.41	0.6	46.57
AGa	Bacteroidales	87	18.00	1.00	54.47
AJ	Bacteroidales	65	6.23	0.03	32
BOa	Clostridiales	51	17.52	0.89	36.17
AIa	Clostridiales	35	10.23	0.67	39.69
AQ	Bacteroidales	35	16.04	0.85	42.73
AFa	Spirochaetales	31	7.55	0.04	37.74
AMa	Spirochaetales	13	7.21	1.66	25.58
APb	Clostridiales	13	13.90	1.85	47.06
AN	Clostridiales	12	5.97	0.95	17.34
AWa	Clostridiales	5	10.84	1.67	30
AS1a	Clostridiales	4	11.46	1.85	27.94
ATa	Clostridiales	2	11.14	2.22	20.05

Table S5. Statistics of the curated database dedicated to the ruminal ecosystem and the number of targeted genera. ssu : small subunit; lsu : large subunit.

		Number of rDNA sequences		Number of genera
		ssu	lsu	
Prokaryotes	Bacteria	18000	4800	179
	Archaea	142	34	10
Eukaryotes	Protozoa	36	3	10
	Fungi	22	27	5

Table S6. Capture probes designed specifically for ribosomal RNA (rRNA) removal from rumen total RNA and the amount added for capture.

**Microorganisms**	**Target**	**Capture probe**	**5'-3' sequence**	**Amount per reaction**
Bacteria	16S rRNA	16Sbact514	HCGTATTACCGCGGCTGCTGGCACG	20 pmol
		16Sbact900	GCTTGTGCGGGYCCCCGTCAATTCC	
		16Sbact780	GCGTGGACTACCAGGGTATCTAATC	
	23S rRNA	23Sbact1050	TGGCTGCTTCYAAGCCAACATCCT	24 pmol
		23Sbact1920	GACAAGGAATTTCGCTACCTTAGGA	
		23Sbact2240	AGTTTGACTGGGGRGGTCGCCTCCT	
Archaea	16S rRNA	16Sarch830	CCCCCGCCAATTCCTTTAAGTTTCA	1 pmol
		16Sarch1004	TCGCTCGTTGCCTGACTTAACAGGA	
	23S rRNA	23Sarch1907	TACCTTAAGAGGGTYATAGTTACCC	1.2 pmol
		23Sarch2572	CACGACGGTCTAAACCCAGCTCACG	
Protozoa	18S rRNA	18Sprot522	CTATTAGAGCTGGAATTACCGCGGC	0.25 pmol
		18Sprot1146	CCACCAACTAAGAACGGCCATGCAC	
	28S rRNA	28Sprot481	CCAATCACACCCTAACCGGCTAAGC	0.3 pmol
		28Sprot178	CACACGCTATACGAGGCTTGCACTC	
Fungi	18S rRNA	18Sfung789	CATTACTTCGGTCCTAGAAACCAAC	0.25 pmol
		18Sfung1060	AGCCTTGCGACCATACTCCCCCCGG	
	28S rRNA	28Sfung638	CTGCAGACCTCAATCTCTCCGATTG	0.3 pmol
		28sfung1941	GGACCGTTAGGAACGCACCGGACAT	

Table S7. Efficiency of the mRNA enrichment method as determined using qPCR and RNA-Seq data.

	RT-qPCR data rRNA copy numbers/µg RNA ± SD			RNA-Seq data rRNA reads (% total initial reads) [1]	
	Before enrichment	After enrichment	rRNA removal (%)	Before enrichment	After enrichment
16S bacteria	$2.83\times10^{10} \pm 6.60 \times10^{9}$	$3.23\times10^{9} \pm 1.51\times10^{9}$	88.78	92.9	85.4
16S archaea	$2.29\times10^{8} \pm 1.69\times10^{7}$	$1.51\times10^{8} \pm 2.63\times10^{7}$	33.77		
18S protozoa	$1.13\times10^{10} \pm 5.64\times10^{9}$	$2.15\times10^{9} \pm 1.15\ 10^{9}$	80.94		
ITS fungi	$1.13\times10^{6} \pm 1.56\times10^{5}$	$4.97\times10^{5} \pm 1.72\ 10^{5}$	54.79		

qPCR was carried out in triplicate on two different RNA extracted from the rumen sample analysed in the present study. Each value is the mean ± standard deviation for n=6 determinations.[1] based on the identification of rRNA with SortMeRNA (Kopylova et al., 2012)

Table S8. Statistics of RNA sequencing and reads trimming, merging, and assembling.

	Total RNA	mRNA enriched RNA
**Paired-end raw reads (n)**	24,282,144	20,922,080
Total size (nt)	4,551,466,369	3,855,176,363
No. of paired-end reads with Q>30	20,746,664	18,145,720
**Merged reads (n)**	9,863,588	8,719,248
Average length (nt)	169	168
No. of rRNA reads	9,163,861	7,450,287
No. of non-rRNA reads	699,727 (7.09%)	1,268,961 (14.55%)
**Contigs (n)**	12,240	23,150
Average length (nt)	354	357
Range length	201-4,442	201-4,616

Table S9. Oligonucleotide primers used for real-time quantitative PCR

Microbial groups	Primer sets	Sequence (5'-3')	Fragment length (bp)	Reference
Total bacteria	520F	AGCAGCCGCGGTAAT	220	Edwards et al. (2007, 2008)
	799R2cor	CAGGGTATCTAATCCTGTT		
Methanogenic archaea	MB1174f	GAGGAAGGAGTGGACGACGGTA	233	Ohene-Adjei et al. (2007)
	Arch1406-1389r	ACGGGCGGTGTGTGCAAG		
Protozoa	P-SSU-316f	GCTTTCGWTGGTAGTGTATT	223	Sylvester et al. (2004)
	P-SSU-539r	CTTGCCCTCYAATCGTWCT		
Neocallimastigomycota	Anaerobic_fungi-F	GAGGAAGTAAAAGTCGTAACAAGGTTTC	120	Denman and McSweeney (2006)
	Anaerobic_fungi-R	CAAATTCACAAAGGGTAGGATGATT		

References

Denman, S. E., and McSweeney, C. S. (2006). Development of a real-time PCR assay for monitoring anaerobic fungal and cellulolytic bacterial populations within the rumen. *FEMS Microbiol. Ecol.* 58, 572–82. doi:10.1111/j.1574-6941.2006.00190.x.

Dugat-Bony, E., Missaoui, M., Peyretaillade, E., Biderre-Petit, C., Bouzid, O., Gouinaud, C., et al. (2011). HiSpOD: probe design for functional DNA microarrays. *Bioinformatics* 27, 641–8. doi:10.1093/bioinformatics/btq712.

Edwards, J. E., Huws, S. A., Kim, E. J., and Kingston-Smith, A. H. (2007). Characterization of the dynamics of initial bacterial colonization of nonconserved forage in the bovine rumen. *FEMS Microbiol. Ecol.* 62, 141–142. doi:10.1111/j.1574-6941.2007.00392.x.

Edwards, J. E., Kingston-Smith, A. H., Jimenez, H. R., Huws, S. a, Skøt, K. P., Griffith, G. W., et al. (2008). Dynamics of initial colonization of nonconserved perennial ryegrass by anaerobic fungi in the bovine rumen. *FEMS Microbiol. Ecol.* 66, 537–45. doi:10.1111/j.1574-6941.2008.00563.x.

Hess, M., Sczyrba, A., Egan, R., Kim, T.-W., Chokhawala, H., Schroth, G., et al. (2011). Metagenomic Discovery of Biomass-Degrading Genes and Genomes from Cow Rumen. *Science (80-.).* 331, 463–467. doi:10.1126/science.1200387.

Kopylova, E., Noé, L., and Touzet, H. (2012). SortMeRNA: fast and accurate filtering of ribosomal RNAs in metatranscriptomic data. *Bioinformatics* 28, 3211–7. doi:10.1093/bioinformatics/bts611.

Larkin, M. A., Blackshields, G., Brown, N. P., Chenna, R., McGettigan, P. A., McWilliam, H., et al. (2007). Clustal W and Clustal X version 2.0. *Bioinformatics* 23, 2947–8. doi:10.1093/bioinformatics/btm404.

Ohene-Adjei, S., Teather, R. M., Ivan, M., and Forster, R. J. (2007). Postinoculation protozoan establishment and association patterns of methanogenic archaea in the ovine rumen. *Appl. Environ. Microbiol.* 73, 4609–18. doi:10.1128/AEM.02687-06.

Pruesse, E., Quast, C., Knittel, K., Fuchs, B. M., Ludwig, W., Peplies, J., et al. (2007). SILVA: a comprehensive online resource for quality checked and aligned ribosomal RNA sequence data compatible with ARB. *Nucleic Acids Res.* 35, 7188–96. doi:10.1093/nar/gkm864.

Sylvester, J. T., Karnati, S. K. R., Yu, Z., Morrison, M., and Firkins, J. L. (2004). Development of an assay to quantify rumen ciliate protozoal biomass in cows using real-time PCR. *J. Nutr.* 134, 3378–84. Available at: http://www.ncbi.nlm.nih.gov/pubmed/15570040.

Tamura, K., Stecher, G., Peterson, D., Filipski, A., and Kumar, S. (2013). MEGA6: Molecular Evolutionary Genetics Analysis version 6.0. *Mol. Biol. Evol.* 30, 2725–9. doi:10.1093/molbev/mst197.
